# A Macro Lens-Based Optical System Design for Phototherapeutic Instrumentation

**DOI:** 10.3390/s19245427

**Published:** 2019-12-09

**Authors:** Hojong Choi, Se-woon Choe, Jae-Myung Ryu

**Affiliations:** 1 Department of Medical IT Convergence Engineering, Kumoh National Institute of Technology, Gumi 39253, Korea; hojongch@kumoh.ac.kr; 2 Department of Optical Engineering, Kumoh National Institute of Technology, Gumi 39253, Korea

**Keywords:** macro lens, light emitting diodes, cancer phototherapy

## Abstract

Light emitting diode (LED) and ultrasound have been powerful treatment stimuli for tumor cell growth due to non-radiation effects. This research is the first preliminary study of tumor cell suppression using a macro-lens-supported 460-nm LED combined with high-frequency ultrasound. The cell density, when exposed to the LED combined with ultrasound, was gradually reduced after 30 min of induction for up to three consecutive days when 48-W DC, 20-cycle, and 50 V_p-p_ sinusoidal pulses were applied to the LEDs through a designed macro lens and to the ultrasound transducer, respectively. Using a developed macro lens, the non-directional light beam emitted from the LED could be localized to a certain spot, likewise with ultrasound, to avoid additional undesirable thermal effects on the small sized tumor cells. In the experimental results, compared to LED-only induction (14.49 ± 2.73%) and ultrasound-only induction (13.27 ± 2.33%), LED combined with ultrasound induction exhibited the lowest cell density (6.25 ± 1.25%). Therefore, our measurement data demonstrated that a macro-lens-supported 460-nm LED combined with an ultrasound transducer could possibly suppress early stage tumor cells effectively.

## 1. Introduction

Phototherapy is a light-based, non-ionizing, anticancer therapeutic treatment, and photodynamic therapy utilizes photosensitizing agents, such as porfimer sodium, lutetium texaphyrin, and SnET2 [1]. A photosensitizer can be activated to localize the tumor in particular tissue regions and these therapies cause tissue death by creating reactive oxygen species and radicals [1]. The mechanism of phototherapy is currently accepted to a mitochondrial electron transport chain that affects photostimulatory effects in a cell [2]. In phototherapy or photodynamic therapy devices, light-emitting diodes (LEDs), capable of generating various wavelengths, have been considered as an alternative light source to fluorescent tube, halogen lamp, and laser beam [3]. LEDs in the visible wavelength (400–600 nm) are applicable as a therapeutic light source to treat skin diseases, cancer cells, and muscle pain because they suppress intercellular signal transduction and metastasis of cancer cells via thermal effects [4,5]. In particular, blue LED irradiation with wavelengths of 400–500 nm has been recently highlighted in various ways, such as to bleach teeth, treat Alzheimer’s disease, reduce muscle pain, and ablate cancer cells [5,6]. Moreover, blue LED light has been reported to suppress melanoma and retinal pigment epithelium cells because it generates intracellular oxygen species that can induce apoptosis without any thermal effects [7,8]. However, the thermal mechanism of the signaling pathways for suppressing cancer cells in blue LED-based phototherapy is still under exploration [9].

Ultrasound transducers have been used as one of the imaging and therapeutic tools for cancer treatment, such as in sonodynamic therapy or ultrasound-mediated chemotherapy [10,11]. The ultrasound transducers used for low-frequency (<15 MHz) ultrasound therapy typically generate mechanical effects, such as cavitation, which leads to the production of free radicals, thus damaging the tissues [12,13]. In addition, sonosensitizer or macrobubble contrast agents can been applied to increase the destruction of malignant cancer cells in these therapies [14]. In ultrasound-mediated chemotherapy, chemotherapeutic agents, such as Adriamycin, cisplatin, and doxorubicin, are utilized to improve the suppression of cancer cell growth by ultrasound insonation [15,16,17]. With the help of sonosensitizers, ultrasound insonation can further help in facilitating the transfection of the nucleic acids in the drug-delivery process, resulting in cancer cell growth reduction or necrosis [18,19,20]. These effects have been confirmed for breast cancer, cervical cancer, and leukemia, but there are several limitations and side effects of sonosensitizers [21,22,23].

The mechanical effects induced by ultrasound transducers can promote tumor necrosis by inducing the appropriate power and frequency of the transmitted sources [24,25,26,27]. Tumor cell structures and functions exposed to low-frequency ultrasound can be deformed, thus resulting in cell damage, necrosis, proliferation, migration, etc. [28,29,30]. Therefore, there is a need to manipulate the control parameters of the inducing sources to obtain suppression effects on the tumor cells. In contrast, high-frequency ultrasound has the potential to be focused onto targeted cells compared to low-frequency ultrasound [31,32,33,34]. However, the threshold conditions to stimulate tumor necrosis or cell suppression using additional photosensitizers with ultrasound are challenging to characterize in in vitro experiments because it is difficult to distinguish between intracellular and extracellular cavitation [35]. Therefore, the suppression effects on tumor cells in our approach were gauged under specific conditions by manipulating the powers at operating frequencies of the ultrasound transducers [36,37,38]. Additionally, models of high-frequency ultrasound make it even more difficult to describe the mechanism of tumor cell inhibition, so there are no unanimous conclusions on the cell membrane mechanism with specific ultrasound stimulation [39,40].

Unfortunately, there are very few research studies using specific LEDs or high-frequency ultrasound transducers for cancer therapy and mechanisms dependent on the power conditions of LED lights with ultrasound transducers to suppress tumor cells [6,9,41]. Our macro-lens-supported LED combined with a high-frequency ultrasound transducer is the first experiment to report on the suppression of the viability of tumor cells without any help of photosensitizers or sonosensitizers. Cell proliferation is one of the important treatment evaluation indexes used in cancer cell treatment studies [42,43]. The HeLa cell has been most widely used for studying tumor cells because its model has been used to characterize numerous aspects of biological processes over 50 years [44]. Our proposed method entailed focusing the LED and ultrasound beam onto the target at a certain spot for a preliminary tumor cell proliferation study without using any additional photosensitizers or sonosensitizers because the regulation of these agents needs to be carefully controlled to have suppression effects on the cancer cells. Macro lenses have a much higher magnification than other optical lenses [45]. The macro lens used in this study had a magnification of −1.0, however the magnification of most other single-focal length lenses does not exceed −0.3 [46]. Additionally, the extension tube was used here to make the imaging magnification larger [47]. In this study, an optical lens was designed so that the magnification could be continuously adjusted up to −1.0. Additionally, in some cases, it was confirmed that even if the magnification was increased −1.3 times by using an extension tube, sufficient resolution was obtained. As magnification increases, the light can be illuminated on the sample with more energy [48]. In this paper, the macro lens with sufficient resolution was designed by adjusting the magnification to −1.0 and using an extension tube.

## 2. Materials and Methods

### 2.1. Macro Lens Design

The LED employed was a noncoherent, nondirectional, and nonmonochromatic light source, so a certain type of optical lens needed to be developed to focus the light beam to avoid additional thermal effects. The focused ultrasound transducer also needed to be controlled in the same way to concentrate the acoustic beam onto the target in a small area of <1 cm.

A macro lens must be capable of focusing from 0 to −1 magnification in the camera field [49]. A zero magnification means that the object is at an infinite distance and a magnification of −1 means that the object is very close. When the LED was placed on the image plane of such a macro lens, the sample was illuminated on the object plane. The light from the LED was focused in a very narrow area by the optical system, which can increase the energy density [50]. This was suitable for our research because the energy density of the light could be controlled for a specific area by focusing the lens. Such a macro lens is divided into a form in which the total length of the optical lens is changed and fixed at the focusing point [51]. If the total length of the optical system is fixed, it is advantageous to construct our experiment. Therefore, in this study, we tried to design a macro lens in which the overall length of the optical system was fixed.

**Figure 1:**
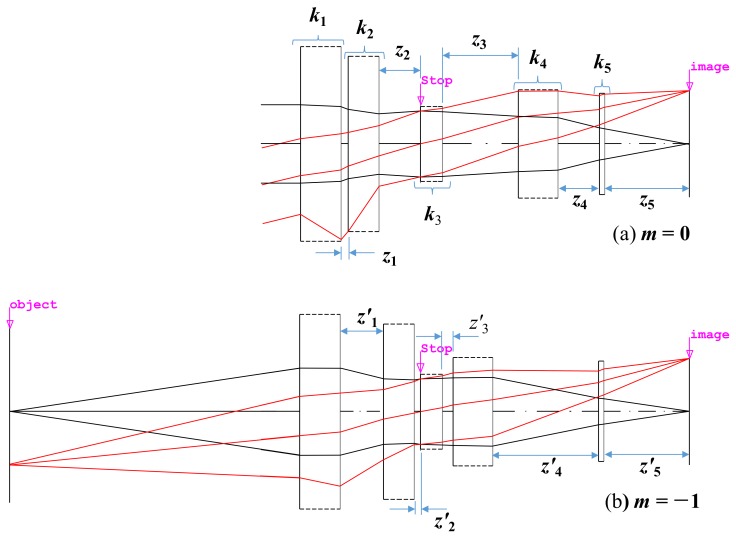
Paraxial layout for our designed optical lens.

The optical lens was composed of five lens groups. In our designed optical lens, two groups needed to be moved to focus on a certain point. At this time, if the total length of the optical lens was fixed, the first and the last lens groups were also fixed. Dust and moisture needed to be prevented from penetrating into this macro lens with a fixed total length. In the optical lens, the stop is normally located in the third lens group, and it is advantageous for the product to be manufactured by fixing the stop. Figure 1 shows the paraxial layout of the designed optical lens to illustrate the path of the light ray; each lens group is represented by an ideal lens group without aberration.

In Figure 1, when the magnification was 0, the paraxial ray tracing for the optical lens is expressed by the Gaussian bracket [52]:

(1) $$\begin{array}{l} \left[k_{1},-z_{1},k_{2},-z_{2},k_{3},-z_{3},k_{4},-z_{4},k_{5},-z_{5}\right]=0\\ \left[k_{1},-z_{1},k_{2},-z_{2},k_{3},-z_{3},k_{4},-z_{4},k_{5}\right]=K \end{array}$$

where *k* is the refracting power of each group, *z* is the distance between the principal points of each lens group, the suffix of each variable is the group number, and *K* is the refracting power of the entire optical lens, which is the inverse of the effective field length (EFL).

Likewise, when the magnification was −1, the paraxial ray tracing for the optical system is expressed as followed by [53]:

(2) $$\begin{array}{l} \left[-{z^{\prime }}_{0},k_{1},-{z^{\prime }}_{1},k_{2},-{z^{\prime }}_{2},k_{3},-{z^{\prime }}_{3},k_{4},-{z^{\prime }}_{4},k_{5},-{z^{\prime }}_{5}\right]=0,\\ \left[-{z^{\prime }}_{0},k_{1},-{z^{\prime }}_{1},k_{2},-{z^{\prime }}_{2},k_{3},-{z^{\prime }}_{3},k_{4},-{z^{\prime }}_{4},k_{5}\right]=-\frac{1}{m}, \end{array}$$

where *z*_0_ is the distance between the object and the first principal point.

Since the distance between each lens group changed, a prime symbol was added to a variable *z*, which indicated a distance between the lens groups. However, since the fifth group must be fixed, *z*_5_ = *z′*_5_ is satisfied. Since the first and third groups must be fixed, *z*_1_ + *z*_2_ = *z′*_1_ + *z′*_2_ and *z*_3_ + *z*_4_ = *z′*_3_ + *z′*_4_ are valid.

In Equations (1) and (2), *K* is determined by the required angle of view of the optical lens, and *m* is −1 since it is the maximum magnification of the optical lens. *K* is the reciprocal value of the focal length of the optical system (*K* = 1/*F*). If the focal length of the optical system is *F*, the angle of view of the optical system is θ, and the size of the sensor is y, then y = *F*·tanθ [54]. Therefore, *K* is determined when the angle of view, which is the optical system photography range, is determined. The macro lens refers to a camera optical system having a maximum magnification of −1. Furthermore, the distance of each lens group can be determined from mechanical conditions appropriately [55]. Therefore, the total number in Equations (1) and (2) is four, and the unknown variables are the refracting power of each lens group. Since the number of unknown variables is larger than that of the Equations (1) and (2), the refracting power of at least one of the five lens groups should be an appropriate value. In addition, if several solutions are given by solving the Equations (1) and (2), then the one with the most physically significant value should be selected.

The initial values of *z*_1_ to *z*_5_ and z′_1_ to *z′*_5_ were input, as shown in Table 1, and *m* was determined to be −1, according to the characteristics of the macro lens. *K* was given from the focal length of the optical lens. For simultaneous use in taking a portrait, the focal length was preferably 60 mm. In addition, if the focal length of the fifth group was 70 mm, the number of unknown variables and the number given by Equations (1) and (2) are then supposed to be equal. Therefore, the solution can be obtained as shown in Table 2. There were a number of methods to obtain solutions by combining Equations (1) and (2). These equations can be solved numerically, such as with the Bairstow or the Newton–Rapson methods [56]. However, the solution can be easily obtained by using mathematical software such as MATLAB program (MathWorks, Natick, MA, USA) [57].

Table 2 shows the focal length of each group. As mentioned earlier, there were several solutions. If the focal length of the third group containing the stop was too short, a surface with a small curvature of each group should be used. Therefore, it could lead to spherical aberration correction. In particular, “solution 3” and “solution 4” were a kind of unrealistic optical lens because the focal length of the entire lens group was too short. Therefore, “solution 1” was the only solution that could be implemented as an optical lens. However, only the focal length of each lens group was obtained in “solution 1”. In order to realize the actual optical lens, the shape of the lens constituting the group had to be determined. In general, it is very challenging to design an entire optical lens including a plurality of lenses. However, it is not difficult to design a bright optical system by constituting three or less lens groups [58]. Therefore, the shape of the optical lens could be determined with the focal length, obtained in Table 2, if the third-order aberration was minimized by the equivalent lens method and the module lens method. In this way, the distances between the lens groups could be determined to satisfy the distance of the principal point, as shown in Table 1. However, even if this method could be applied, desired optical resolution performance would not be obtained. Therefore, the optical aberration had to be minimized through the optimization process.

**Figure 2:**
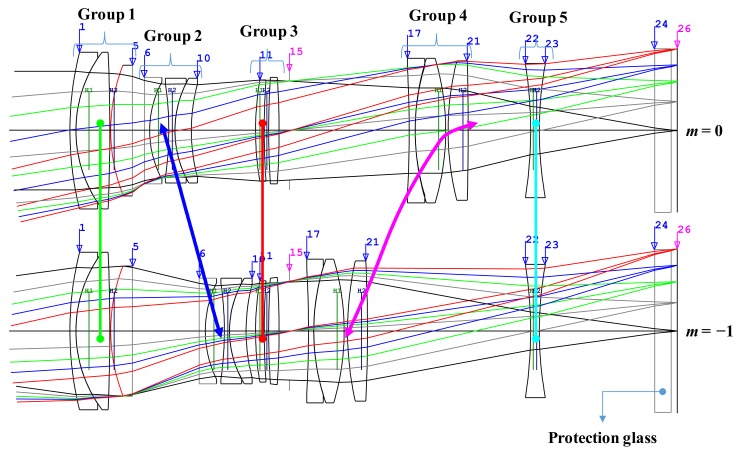
Optical layout for our optimized macro lens.

The layout of the final optical lens is shown in Figure 2. In this figure, the upper optical layout is shown when the object was at infinity and the bottom is when the magnification was −1. The thick line connecting each lens group represents the locus of the lens group when the magnification of the optical lens was changed. The first group, the third group, and the fifth group are described as being fixed, and the thick line connecting the fixed lens group is shown as a straight line.

**Figure 3:**
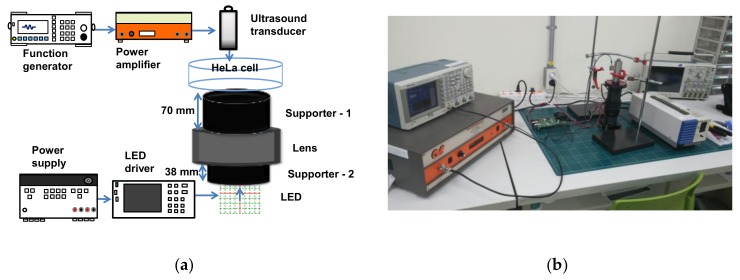
(**a**) Experimental concept and (**b**) setup of the macro-lens-supported light-emitting diode (LED)-ultrasound transducer.

**Figure 4:**
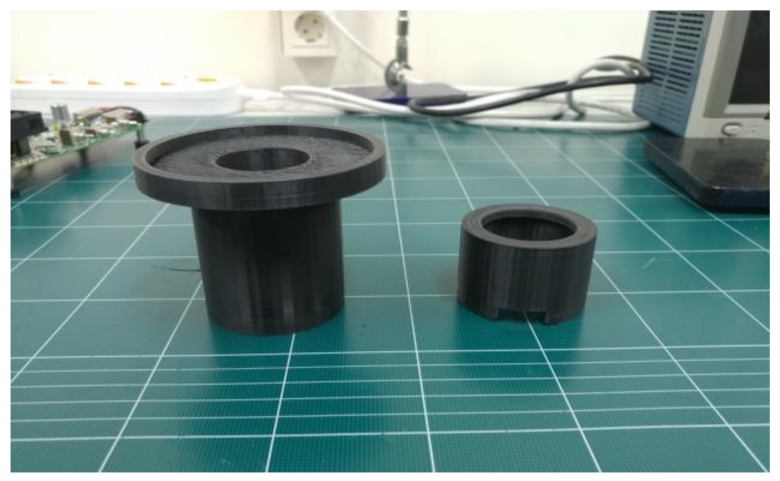
Photograph of support-1 (left) and support-2 (right).

Figure 3 shows the experimental diagram of the macro-lens-supported LED combined with the transducers and the HeLa cell on the petri dish. The custom-made plastic structures were fabricated by a three-dimensional printer (Cubicon 3DP-310F, High Vision System, Seongnam, Republic of Korea) and two supports (support-1 and support-2) were composed of upper (supporter-1) and bottom (supporter-2) parts, which were located between the LED lights and lens and the lens and petri dish, respectively. As shown in Figure 4, supporter-1 was designed to support the petri dish, which sometimes needed to be separated to check the cell viability on the optical macroscope, and supporter-2 was designed to have top and bottom opening spaces to connect the LED power line and not to interrupt LED irradiations, respectively. Moreover, these supports increased the distance between the LED and the petri dish to reduce heat.

### 2.2. Experiment Setup

HeLa cells (Korean Cell Line Bank, Seoul) were cultured in high-glucose Dulbecco’s modified eagle medium (DMEM) containing 10% fetal bovine serum (FBS) and 1% penicillin streptomycin. The prepared cells were incubated at 37 °C in a humidified incubator at 5% CO_2_. The cells were trypsinized when 80% confluence was achieved, washed three times with phosphate buffer solution (PBS), and re-suspended at a density 5 × 10^5^ cells in a 90-mm diameter petri dish. When the cell confluency reached about 40% in the petri dish, the LED/ultrasound stimuli were administered and cells counted everyday up to day 3. The ambient temperature was adjusted to 26 °C to avoid thermal damage to the cells.

A 50 V_p-p_ and 20-cycle sinusoidal pulse signal with a 1 kHz pulse repetition period was generated by a high voltage amplifier (75A250A) and was then sent to the 0.25 in diameter, 0.5 in focused ultrasound transducer. The ultrasound signals were generated by the transducer in order to be directly focused into the cells at the correct focal distance. Then, 48 W power, generated by DC power supply (PAS20-36, Kikusui Electronics Corp., Yokohama, Japan), was sent to the LED-supporting driver (DK-136M, Luminus Devices, Sunnyvale, CA, USA) to produce a 460 nm LED light with the specially-designed macro lens combined with custom-made plastic structures. Acoustic power is typically proportional to the excitation voltages generated from the power amplifiers [59,60]. The acoustic power of a high-frequency ultrasound transducer less than 60 MHz can be measured using an acoustic hydrophone [61]. However, there is no concrete method for obtaining the acoustic pressure of an ultrasound transducer operating over 60 MHz [39,62]. Therefore, we measured pulse-echoes, which is a typical method for evaluating the performances of ultrasound transducers [63,64,65]. The measured condition of the obtained echo signal was the same as the stimulation condition. After the power amplifier (75A250A), the 50 V_p-p_ and 20-cycle sinusoidal pulse signals with a 1 kHz pulse repetition period were 29.57 mV_p-p_.

**Figure 5:**
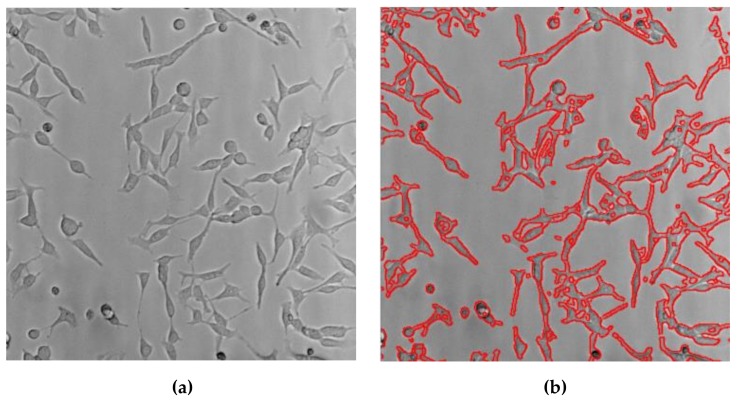
Representative images of cell density measurement. (**a**) Acquired brightfield image and (**b**) cell boundary image traced by red colored solid lines.

The LED with macro lens under the petri dish and ultrasound transducer were placed, as shown in Figure 3, in order to focus the light and acoustic powers into the HeLa cell samples in the petri dish simultaneously. In the literature, Ca^+^ ion response was shown when ultrasound stimulation was induced for at least 10 or 20 min [66]. In this experiment, LED light, ultrasound signals, or LED light combined with ultrasound signals stimuli were transmitted into the HeLa cells for 30 min daily up to 3 days and Hela cell images were acquired using optical microscopy. Afterwards, survived cell density was quantified using multiple image processing techniques and statistical analysis was performed to compare each experiment by using MATLAB software (MathWorks, Natick, MA, USA), as previously described in [67]. All brightfield images of the HeLa cells were acquired on an inverted macroscope to find and compare optical results in each experimental method. Since the stimulation procedure was initiated when the cell confluency reached about 40% in the petri dish, any perceptible difference in cell concentrations of each group was observed once a day. Five randomly chosen brightfield images of each petri dish were acquired every day by inverted microscope and cell concentration was determined after completion of individual stimulus induction to each group. The cell density was normalized to the cell number in the control group and compared to the experimental groups on the same day. Fifty brightfield images were obtained to quantify the cell density per group (*n* = 10/group), so that a total of two hundred images were used for all four groups including the control, LED alone, ultrasound (US) alone, and the LED+US group. Individual samples in each group were independently analyzed and repeated ten more times under the same experimental protocol. Briefly, all brightfield images were obtained over the same area where each stimulus was applied in a petri dish. To enumerate cell densities, the obtained brightfield microscopic images on the same area were taken immediately after stimuli induction. Various image processing techniques, including the adaptive thresholding method, were applied to segment and quantitatively analyze survived cells density of each group. The cell densities were calculated by dividing the pixel number of the survived cell grown area by the obtained microscopic pixel number. Figure 5 shows example images used for quantitative analysis using an acquired brightfield image (Figure 5a) and post-processed image with red colored cell boundary (Figure 5b). Analysis of variance (ANOVA) with Scheffe’s post hoc test was applied to assess the differences between the control and experimental groups and *p* values of less than 0.05 were considered statistically significant.

## 3. Results and Discussion

### 3.1. Performance Verification of Macro Lens

The maximum magnification of the optical lens designed in our research was −1. This means that the area illuminated was equal to the size of the LED. To illuminate a narrower area, the magnification of the optical lens must be increased [68]. On the other hand, the imaging equation of the optical lens with object distance *L*, image distance *L*′, and EFL is *F* is expressed as follows:

(3) $$\frac{1}{L}+\frac{1}{L^{\prime }}=\frac{1}{F}.$$

Since the magnification is *m* = −*L’*/*L*, *L′* must be large in order to increase *m*. Therefore, we used this principle to increase the magnification of the macro lens. The magnification *m* can be expressed as *L′* and *F* as shown in Equation (4):

(4) $$m=1-\frac{L^{\prime }}{F}.$$

If the image distance increases by the length change ∆*L′*, the magnification change ∆m can be calculated as shown in Equation (5):

(5) $$\Delta m=-\frac{\Delta L^{\prime }}{F}.$$

**Figure 6:**
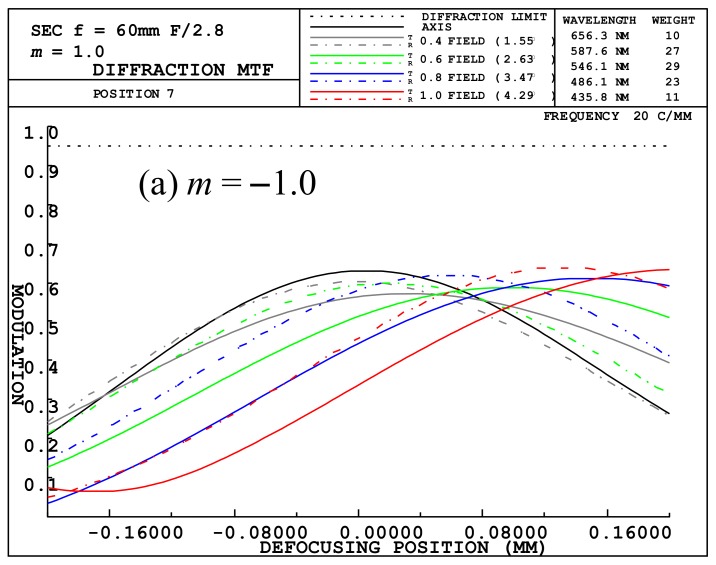

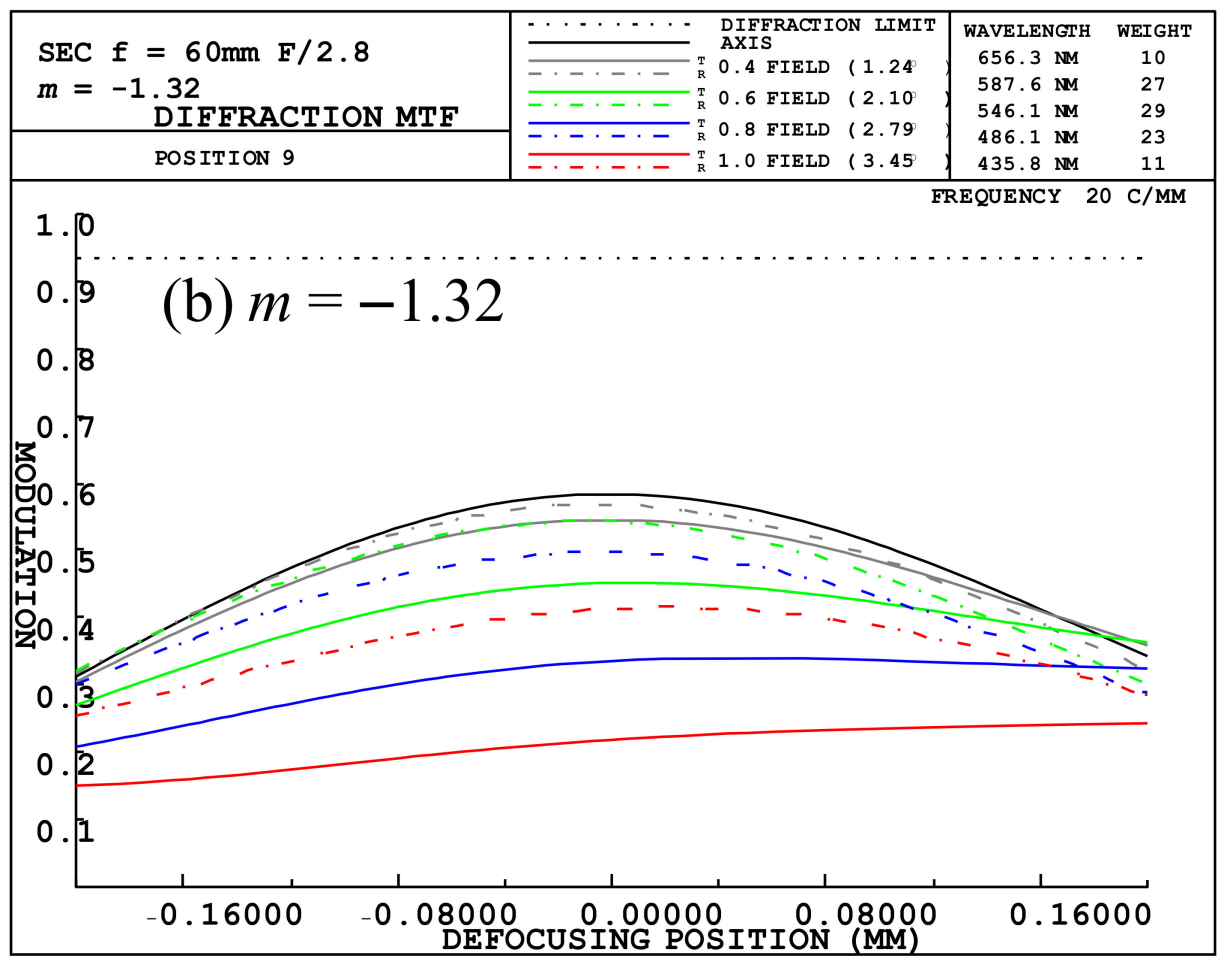
Defocused modulation transfer function (MTF) plots for our designed macro lens at (**a**) *m* = −1.0 and (**b**) *m* = −1.32.

When the magnification was −1, the focal length of the optical lens was about 39.015. If the image distance was increased by −12.5 mm, the magnification increased by about 0.32 times, using Equation (5), resulting in a magnification of −1.32. *m* = −1 is the value given when using the macro lens, and *m* = −1.32 indicates a magnification used to decrease the illumination area. *m* = −1.32 can be achieved by increasing the back focal length (BFL) of the optical system. The modulation transfer function (MTF) of the designed optical system was about 0.58 (*m* = −1.32) to 0.62 (*m* = −1) at the center, as shown in Figure 6. The MTF of the designed optical system was about the same as that of macro lens made by other companies. However, optical resolution performance of the lens was considered only when the magnification was −1. When magnification increased by ∆*m*, it was necessary to confirm whether there was a problem for obtaining resolution performance. A typical physical quantity for determining optical resolution performance is a modulation transfer function (MTF) [54]. Figure 6 shows the defocused MTF plots of our designed macro lens. The magnification is −1.0 on Figure 6a and −1.32 on Figure 6b.

Figure 6 illustrates a spatial frequency of 20 LP/mm with a minimum resolution of 25 μm. Since the optical lens was designed with a maximum height of 14.25 mm, it was not a big problem to use an LED with a size of 4.6 mm × 2.6 mm at the same performance, as shown in Figure 6. Since this was about 1/100 of the vertical length of our used LED, the sharpness of the illumination area was not a problem. In order to focus the light to a smaller spot, the LED was positioned at 12.5 mm above the focal plane.

**Figure 7:**
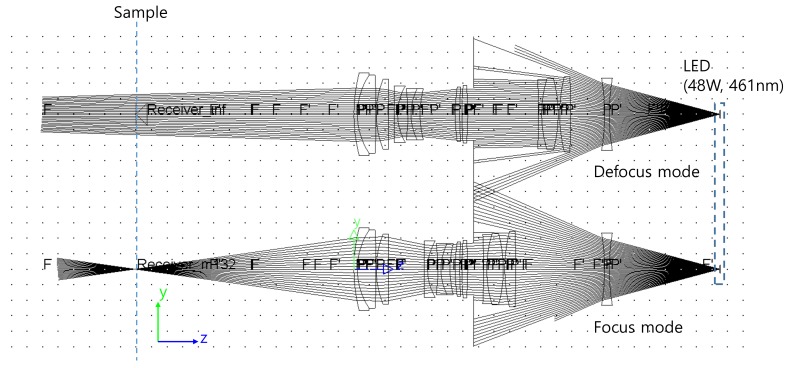
Layout of the optical rays in the designed Macro lens.

Figure 7 shows the input results of the illumination distribution. When the lens group was located at *m* = 0, as shown in Figure 4, it can be seen that the light was not well focused on the sample surface. However, the light was well focused at *m* = −1 for our designed macro lens. This allowed the energy density on the sample to be adjusted. Therefore, “defocus mode” is the case where light was not focused well and the energy density was low, and “focus mode” is the case where light was condensed and energy density was high. When the LEDs were 48 W and 461 nm, the results of the optical rays in the macro lens are shown in Figure 7. The defocus mode showed a circular shape with a diameter of about 13 mm and a light distribution with a 3.47 mm × 1.96 mm rectangular shape with LED reduced by 1.32 times in the focus mode. At the center of the illuminated distribution, the intensity of light was 6.4 mW/mm^2^ and 81.34 mW/mm^2^ in the defocus and focus modes, respectively.

### 3.2. In Vitro HeLa Cell Experiments with Designed Instrumentation

In order to examine the suppression effect of stimuli, we divided samples into 4 different groups (control without ultrasound (US) or LED, US only, LED only, and LED+US stimuli (*n* = 10/group)). No stimuli were induced in the control group (*n* = 10) but the other groups were exposed to 460 nm LED or US transducer, and LED combined with the transducer for 30 min up to 3 days. Even though the thermal and mechanical effects caused by 460 nm LED and ultrasound are still under research, LED and ultrasound devices could effectively reduce cancer cell viability with signaling effects to the cells and cavitation effects [5,25]. For LED, light irradiation inhibits the signaling between cancer cells, thus inducing cell death without thermal increments [9]. For ultrasound, cavitation effects are produced in the tissues to collapse gaseous cavities in a medium.

**Figure 8:**
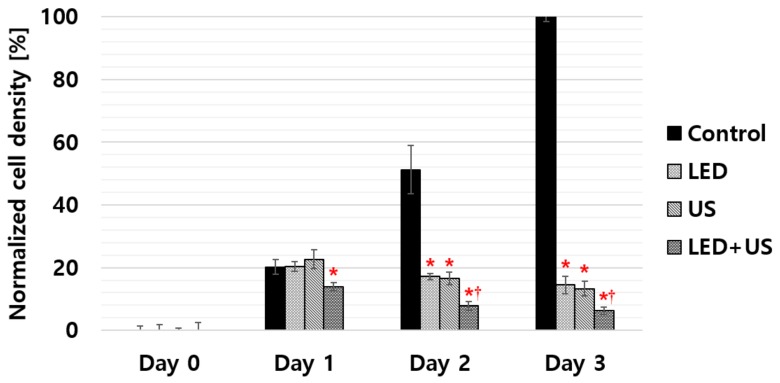
Selective experimental results (*p < 0.05,^†^*p* < 0.01) of the cell densities when using the LED, the US transducer, and the LED+US transducer up to day 3. The black bar represents the control group of HeLa cells.

All the enumerated cell densities are represented as mean ± standard deviations in Figure 8 and Table 3. According to our experimental results, 460 nm LED alone, US alone, and LED+US without additional chemotherapeutic agents can inhibit cell proliferation. Only the LED+US group started to show statistical significance compared to the control group from day 1. However, all the experimental groups (LED alone, US alone, and LED+US) showed statistical significance compared to the control group from day 2 to the last day of the experiment. Especially, the LED+US stimulus showed the lowest *p* value (<0.01) when compared to LED or US alone, whereas statistical significance was not observed between LED alone and US alone on the same day. Table 3 summarizes the experimental data of the HeLa cell density when using LED, US and LED+US including control group. As shown in Table 3, on day 3, the normalized cell densities of the control, LED, US, and LED + US groups were 100 ± 1.40%, 14.49 ± 2.73%, 13.27 ± 2.33%, and 6.25 ± 1.25%, respectively. Our experimental results confirmed that cell densities were significantly suppressed when LED, US, and 460 nm LED + US stimuli were exposed to the HeLa cells compared to control group on day 3. There were no noticeable cell density changes between the LED and US groups from days 1 to 3, but the LED + US stimulus showed the dramatic suppression of the cell proliferation rate and statistically meaningful changes from days 1 to 3.

## 4. Conclusions

Ultrasound and LED are non-ionizing treatment sources. We proposed the used of LED using a macro lens combined with a transducer to possibly ablate HeLa cells without the use of photosensitizers or sonosensitizers. It is because the macro lens could cause cell suppression by focusing the divergent LED lights to produce focused light spots. The ultrasound was also an effective treatment solution to focus on the small spots.

Based on the best of our knowledge, we firstly propose macro-lens-supported LED combined with a transducer and without any therapeutic agents to suppress HeLa cell proliferation. Our experimental results demonstrated that LED, ultrasound, and LED combined with ultrasound stimulus without additional chemotherapeutic agents can suppress cell proliferation. The experimental results, including the control and experimental groups (LED, US, and LED+US), were 100 ± 1.40%, 14.49 ± 2.73%, 13.27 ± 2.33%, and 6.25 ± 1.25% on day 3. Compared to LED or ultrasound alone, LED combined with ultrasound stimulus caused the lowest cell viability. Therefore, we demonstrated that our tumor cell suppression approach with LED using a macro lens and ultrasound transducer is more effective than LED or ultrasound alone to reduce tumor proliferation. We believe that experimental results using LED combined with an ultrasound transducer may also be combined with other cancer suppression techniques, such as chemotherapy, photodynamic therapy, etc.

## Figures and Tables

**Table 1:** Initial distance between each group.

	*z*_1_	*z*_2_	*z*_3_	*z*_4_	*z*_5_
*m* = 0	7.5	15.5	30	12.5	25
*m* = −1	17.5	5.5	12.5	30	25

**Table 2:** Result of power arrangement.

	Solution 1	Solution 2	Solution 3	Solution 4
*f*_1_	32.31812	29.67649	2.302914	8.615394
*f*_2_	−20.752	−36.0855	3.875917	5.64389
*f*_3_	75.08157	−30.1579	0.297674	8.811696
*f*_4_	40.65758	25.21832	15.65266	6.165469

**Table 3:** Normalized data for the experimental cell density.

	Day 0	Day 1	Day 2	Day 3
Control	0.00 ± 1.44	20.24 ± 2.35	51.26 ± 7.69	100.00 ± 1.40
LED	0.00 ± 2.35	20.36 ± 1.51	17.19 ± 0.96	14.49 ± 2.73
US	0.00 ± 0.79	22.64 ± 3.05	16.52 ± 1.99	13.27 ± 2.33
LED+US	0.00 ± 2.57	13.88 ± 1.42	7.91 ± 1.34	6.25 ± 1.25
